# SSUnique: Detecting Sequence Novelty in Microbiome Surveys

**DOI:** 10.1128/mSystems.00133-16

**Published:** 2016-12-20

**Authors:** Michael D. J. Lynch, Josh D. Neufeld

**Affiliations:** Department of Biology, University of Waterloo, Waterloo, Ontario, Canada; Northern Arizona University

**Keywords:** 16S rRNA, high-throughput sequencing, microbial dark matter, microbiome, rare biosphere, taxonomic blind spots, taxonomic novelty

## Abstract

High-throughput sequencing of small-subunit (SSU) rRNA genes has revolutionized understanding of microbial communities and facilitated investigations into ecological dynamics at unprecedented scales. Such extensive SSU rRNA gene sequence libraries, constructed from DNA extracts of environmental or host-associated samples, often contain a substantial proportion of unclassified sequences, many representing organisms with novel taxonomy (taxonomic “blind spots”) and potentially unique ecology. Indeed, these novel taxonomic lineages are associated with so-called microbial “dark matter,” which is the genomic potential of these lineages. Unfortunately, characterization beyond “unclassified” is challenging due to relatively short read lengths and large data set sizes. Here we demonstrate how mining of phylogenetically novel sequences from microbial ecosystems can be automated using SSUnique, a software pipeline that filters unclassified and/or rare operational taxonomic units (OTUs) from 16S rRNA gene sequence libraries by screening against consensus structural models for SSU rRNA. Phylogenetic position is inferred against a reference data set, and additional characterization of novel clades is also included, such as targeted probe/primer design and mining of assembled metagenomes for genomic context. We show how SSUnique reproduced a previous analysis of phylogenetic novelty from an Arctic tundra soil and demonstrate the recovery of highly novel clades from data sets associated with both the Earth Microbiome Project (EMP) and Human Microbiome Project (HMP). We anticipate that SSUnique will add to the expanding computational toolbox supporting high-throughput sequencing approaches for the study of microbial ecology and phylogeny.

**IMPORTANCE** Extensive SSU rRNA gene sequence libraries, constructed from DNA extracts of environmental or host-associated samples, often contain many unclassified sequences, many representing organisms with novel taxonomy (taxonomic “blind spots”) and potentially unique ecology. This novelty is poorly explored in standard workflows, which narrows the breadth and discovery potential of such studies. Here we present the SSUnique analysis pipeline, which will promote the exploration of unclassified diversity in microbiome research and, importantly, enable the discovery of substantial novel taxonomic lineages through the analysis of a large variety of existing data sets.

## Abstract (precis)

Extensive SSU rRNA gene sequence libraries, constructed from DNA extracts of environmental or host-associated samples, often contain many unclassified sequences, many representing organisms with novel taxonomy (taxonomic “blind spots”) and potentially unique ecology. This novelty is poorly explored in standard workflows, which narrows the breadth and discovery potential of such studies. Here we present the SSUnique analysis pipeline, which will promote the exploration of unclassified diversity in microbiome research and, importantly, enable the discovery of substantial novel taxonomic lineages through the analysis of a large variety of existing data sets.

## INTRODUCTION

High-throughput sequencing provides insight into the enormous microbial diversity of global ecosystems, highlighting a substantial diversity of new microbial species. Increases in sequencing capacity also led to the recognition of microbial taxa present at low relative abundance, which has been termed the “rare biosphere” (1, 2) and is often a main contributor to local species richness. Previous work has attempted to target low abundance and/or phylogenetic novelty directly, including work in hot springs (3) and Arctic tundra (4), which are both generally poorly characterized environments harboring considerable unknown diversity. Identification of phylogenetic novelty has also been used to address unknown blind spots in reference data sets (5). Even seemingly well-studied environments have a large proportion of unclassified taxa, suggesting a prevalence of considerable uncharacterized microbial diversity and arguing for a large-scale investigation into the novelty of microbial ecosystems across all biomes. This is a first step in further addressing hypotheses related to uncharacterized microorganisms, reflected by searches for the fourth domain (6) and microbial dark matter (7). Investigations into uncharacterized microbial diversity can also inform bioprospecting (8, 9) and can help with the design of unique probes useful for targeted single-cell genomics (e.g., see reference 7), vastly increasing the types of genomes currently sequenced.

One major limitation to the recovery and characterization of sequences from novel microorganisms is their efficient identification from large sequence data sets. Unclassified sequences are often not further evaluated in 16S rRNA gene studies, and efforts to target novelty have been limited to manual analyses, typically employing individual primer design (3, 4) or resource-intensive cell screening and single-cell genomics (7). Here we demonstrate the utility of SSUnique, a computational tool for exploring, visualizing, and characterizing the unclassified fractions of 16S rRNA gene data sets, specifically highlighting the phylogenetic novelty observed relative to reference data sets. This clade-based approach results in a 2-fold benefit by highlighting reproducible unclassified diversity represented as monophyletic operational transcriptomic units (OTUs) and by establishing a putative evolutionary context for targeted sequences. We demonstrate the effectiveness of this automated pipeline against a previous manual analysis of phylogenetic novelty and the rare biosphere (4) and explore existing uncharacterized phylogenetic novelty through mining of extensive microbiome data sets from the Earth Microbiome Project (EMP) and the Human Microbiome Project (HMP). We discovered phylogenetic novelty across multiple distinct biomes and body sites with this initial SSUnique survey. Our objective is to better characterize the phylogenetic breadth within existing unclassified microbiome sequence data and to direct future exploration of the bacterial tree of life and microbial dark matter through characterization of taxonomic blind spots identified by phylogenetic novelty, frequently overlooked by existing methodologies.

## RESULTS AND DISCUSSION

### Description and impact of the SSUnique pipeline.

SSUnique is an analysis pipeline for exploring phylogenetic novelty in microbiome surveys. The goal is to identify monophyletic groups of unclassified operational taxonomic units (OTUs) in microbiome survey data, characterize observed phylogenetic novelty and potential genomic context, and provide data for downstream analyses. Broadly, survey data are screened for unclassified OTUs that still conform to 16S/18S rRNA gene models. These filtered sequences are merged with default or user-specified reference data, constructing a reference seeded phylogeny used to identify and rank clades of unclassified OTUs. Various additional outputs are provided, including alignment models of each novel clade and visualizations of phylogenetic novelty, especially useful for exploring very large data sets. See Materials and Methods for further details.

Amplicon sequencing of microbiomes provides unprecedented access to the distribution and abundance of species in the environment. With the magnitude of data generated routinely for microbiome analysis, there exists a significant amount of unanalyzed phylogenetic diversity that is frequently ignored. This is unsurprising because characterization of novelty is infrequently related to the hypotheses being studied. However, incorporation of novelty screens into standard workflows would rapidly accelerate phylogenetic and taxonomic research in microbiology and facilitate studies of microbial dark matter. SSUnique represents a rapid and broad approach to further identify and characterize novel microbial diversity associated with amplicon sequencing projects.

### Phylogenetic novelty in Alert, NU, soils.

**FIG 1:**
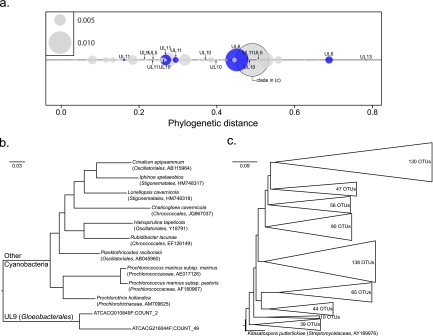
Phylogenetic novelty observed in the Alert, NU, Illumina library (42) showing (a) the recovery of unique lineages (UL) observed in a manual survey for phylogenetic novelty in the same library (4), including (b) a novel clade corresponding to a group of recovered UL examples, and (c) an abundant putatively novel clade not previously observed.

In order to test SSUnique, we performed an automated analysis of phylogenetic novelty on a small subunit (SSU) rRNA gene library from an Alert, Nunavut (NU), Canada, soil sample that was previously analyzed manually for novel clades (4). In that previous research, we targeted groups of unclassified V3 sequences for further characterization, identifying up to 13 unique lineages (ULs). The automated analysis here identified a total of 528 novel clades, 252 consisting of a single OTU; 414 clades persisted after removal of OTUs contributing fewer than 10 sequences (Fig. 1), including a majority of the single-OTU clades. Previously recovered novel clades, including both the short read and corresponding near full-length sequences, were predominantly ranked in the top quartile in the SSUnique output, including the top two ranked clades, which corresponded to UL6 and UL13 (https://github.com/neufeld/SSUnique/blob/master/supplemental.tar.gz). SSUnique was more selective than the previous manual analysis because it excluded the putatively novel clade that did not result in a novel lineage after targeted amplification (UL4). Additionally, some previously identified novel lineages (e.g., UL6, UL10, and UL11) were broadly distributed across the bacterial tree when amplified near-full-length sequences were analyzed (4). These clades were each separately recovered in this analysis (Fig. 1). The *Gloeobacterales* clade, which was highlighted in the previous study (UL9 [4]), was similarly identified here (Fig. 1b) but not highly ranked (172 of 414 novel clades), likely due to lower relative novelty of the V3 region in this amplicon relative to the near-full-length SSU sequence and its moderately low abundance in the data set.

In addition to recovering novelty observed previously, SSUnique highlighted many more novel clades in the Alert, NU, sequence library. One abundant clade was also one of the most novel groups observed in the data set (Fig. 1c). This OTU-rich clade was divergent from known reference sequences, but placed sister to *Kitasatospora putterlickiae*, a member of the *Streptomycetaceae* that was isolated from rhizosphere soil of a *Putterlickia verrucosa* plant from South Africa (10). SSUnique, specifically using clade-based novelty identification rather than sequence identity and BLASTn analysis, more clearly identified cohesive novel groups with multiple OTUs. This resulted in novel clades with more OTUs and typically higher abundance than those highlighted in a manual analysis of the same data (4). For example, seven novel clades each contributed greater than ~0.5% relative abundance, and one of them contributed >1% of total sequence abundance (Fig. 1c).

SSUnique recovered phylogenetically novel and contextually relevant clades from extensive microbiome databases. Manual processing of such data from a previous study was confirmed (4), and this study identified additional novel clades that correspond to several candidate taxa, e.g., independently recovering OTUs corresponding to the recently proposed candidate phylum GH02 (5). The identification of contextually relevant clades, ranked highly by SSUnique, reinforces the utility of this automated analysis pipeline for identification and characterization of phylogenetic novelty in microbiome data.

### Phylogenetic novelty in microbiome databases.

Using Earth Microbiome Project (EMP) data, unassigned taxa represented a larger proportion of microbial richness in diverse and less-studied environments (e.g., terrestrial, forested sites), in contrast to human or animal microbiomes. Specifically, 19.4% (human-associated) to 62.5% (terrestrial) of unweighted OTUs from EMP data and 0.8% from the Human Microbiome Project (HMP) library, which had lower sampling depth and more consistent OTU construction, were not classified to class. A substantial fraction of OTUs in the EMP data, representing >10% of unclassified OTUs in some samples (3.8 to 46.5%; mean, 12.7% [https://github.com/neufeld/SSUnique/blob/master/supplemental.tar.gz]), corresponded to non-SSU sequencing artifacts that did not align to the structural model. These sequences were predominantly associated with PhiX (Illumina sequencing control for low-diversity samples—e.g., 16S rRNA amplicons) and other non-SSU sequences not eliminated from data sets before EMP submission. These artifacts were removed within the SSUnique pipeline by binning OTU sequences by aligning to the bacterial SSU structural model using ssu-align (11). Similar binning can be used to investigate archaeal and eukaryal subsets given appropriate sequencing data.

**FIG 2:**
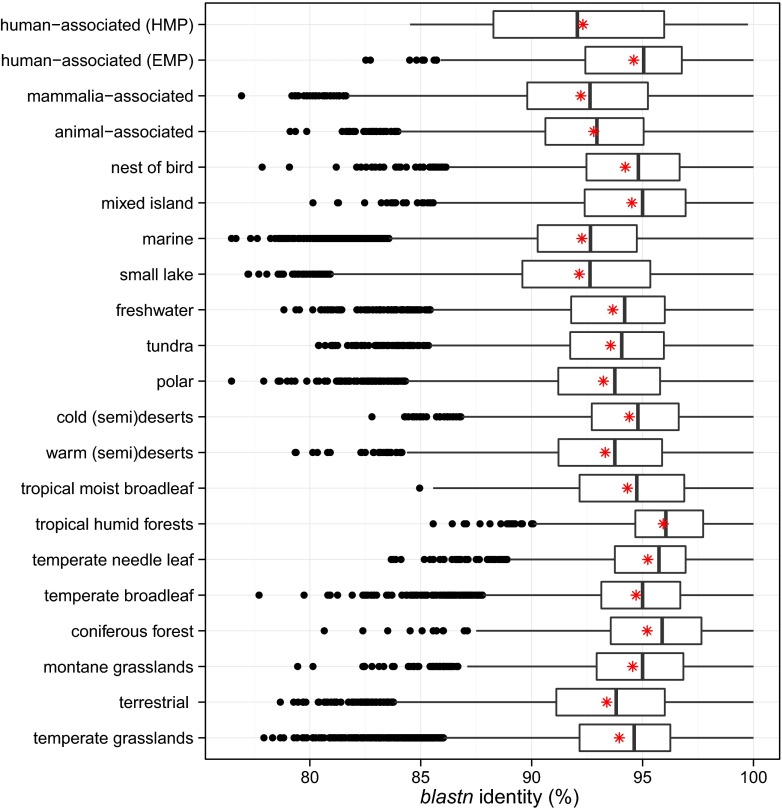
Sequence novelty among SSUnique pipeline-filtered OTUs (unclassified at the class level) in the Earth Microbiome Project (EMP) and Human Microbiome Project (HMP) data, identified by BLASTn search of a 10% random subset against nonredundant NCBI GenBank (excluding environmental and uncultured sequences). Asterisks correspond to mean sequence BLASTn identity.

The mean BLASTn sequence identity to GenBank (nonenvironmental) for representative sequences from a 10% subset of novel OTUs for each biome type was ~94%, with each biome library containing OTU sequences with very low BLASTn identity (Fig. 2). Marine, polar, and temperate grasslands had the highest number of representative sequences with low BLASTn identity (<80%). Biomes with fewer samples and lower sampling effort tended to have fewer OTU sequences with low BLASTn identity (e.g., tropical moist broadleaf and tropical humid forests with 10.9% and 1.4% of OTUs at <90% identity, respectively). In contrast, low-identity OTUs were largely absent from human-associated EMP and HMP biomes, despite a large number of samples. Animal-associated and mammal-associated samples were also substantially more divergent than either human-associated library (EMP or HMP).

General trends of phylogenetic novelty were maintained when correcting for both sequencing and sampling intensity (normalizing weighted phylogenetic distance by either number of samples or number of sequences). Human-associated samples had consistently much less novelty than animal-associated or environmental sites and forested biomes (tropical and subtropical forests, temperate needleleaf and broadleaf forests), and tundra sites contained the highest novelty. Not surprisingly, some samples with particularly low sampling intensity (e.g., coniferous forest biome, 19 samples) had highly variable ranking after normalization.

### Phylogenetic novelty in EMP data.

Phylogenetic novelty was skewed toward ecosystems that were previously known to harbor complex or understudied microbial communities, such as tundra, cold ecosystems, forests, and some aquatic habitats (e.g., freshwater). The number of novel clades observed in these analyses precluded a careful characterization of the entire output of SSUnique; however, the most abundant and novel clades were largely contextually relevant (i.e., made ecological sense). Such ecological consistency, as well as clade membership distributed across multiple experiments and facilities, supports the legitimacy of these observed novel lineages, although in some cases, the closest reference sequences were diverse enough to limit ecological inference.

**FIG 3:**
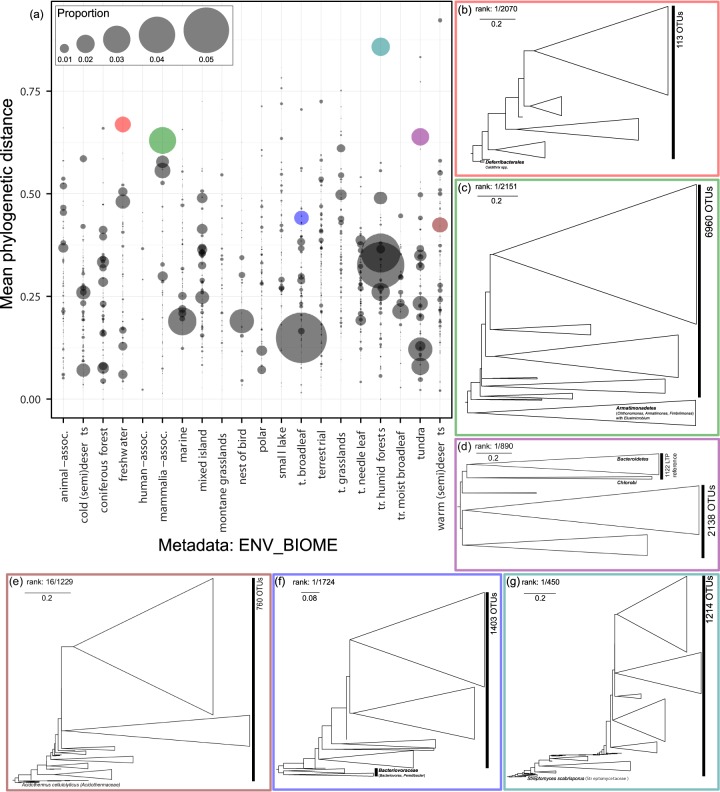
Phylogenetic novelty in biomes represented within Earth Microbiome Project (EMP) data. Each point in panel a represents a clade containing OTUs that were not classified to class, delimited by the nearest known taxonomic reference (Living Tree Project v.119). Point size corresponds to the proportion of sequences in the library grouped within the identified clade. Point colors correspond to colored boxes surrounding phylogenetic trees (b to g), each highlighting selected notable clades discussed in the text. Phylogenetic distance is defined as the mean branch length between each novel terminal node and the nearest taxonomic reference. t., temperate; tr., tropical.

Novel clades in EMP data typically had mean weighted branch lengths of <0.5 substitution per site to known Living Tree Project (LTP) seed sequences (Fig. 3). These clades appeared to be generally distributed across all environment types and novelty was associated with sequencing effort, such that filtered library size was positively correlated with phylogenetic novelty (*r* = 0.8). Cold samples, such as cold deserts, semideserts, and tundra, contained substantial phylogenetic novelty despite lower sampling effort (see Table S1 in the supplemental material). For example, samples from polar environments had a high degree of phylogenetic novelty despite a low number of samples (Fig. 3a). Forested sites also had a large number of novel clades, some being abundant and OTU rich. Human sites demonstrated the lowest phylogenetic novelty, consistent with the lower proportion of unclassified sequences (ca. 19% unclassified at the level class). In contrast, samples characterized as animal associated and *Mammalia* associated harbored substantially more phylogenetic novelty than human samples.

10.1128/mSystems.00133-16.1 Table S1  Marker gene libraries and associated number of samples analyzed in this study. Download Table S1, PDF file, 0.1 MB. Copyright © 2016 Lynch and Neufeld. 2016 Lynch and Neufeld This content is distributed under the terms of the Creative Commons Attribution 4.0 International license.

### Aquatic ecosystems.

Freshwater sites harbored fewer novel clades than marine sites, but contained the most abundant and phylogenetically novel clades of any aquatic biome (Fig. 3a). For example, one divergent freshwater clade corresponded to ~1.7% relative abundance. This clade’s closest reference group contained *Caldithrix* spp. (Fig. 3b), specifically *Caldithrix abyssi*, a nitrate-reducing thermophile from a hydrothermal vent (12). In contrast, the marine biome contained the largest number of novel clades (4,189 clades [3,749 when excluding OTUs with fewer than 10 sequences]), although few clades were of substantial abundance, each representing low to moderate novelty. Divergent clades, those with a mean distance of more than 0.25, each accounted for much less than 1% relative abundance in the marine biome. The largest novel marine clade, not highly ranked by the automated pipeline (https://github.com/neufeld/SSUnique/blob/master/supplemental.tar.gz), had the closest LTP references from marine species belonging to the newly established *Sphingorhabdus* genus (13): *S. flavimaris*, *S. marina*, and *S. litoris*.

### Animal-associated ecosystems.

Three biome subsets from the EMP data contained samples associated with animals: animal (e.g., fish gills, animal feces, including mammals, Komodo dragons, birds, and animal skin surface), humans (various body sites), and *Mammalia* associated (feces from various mammal species). Even though these were three of the top four most sampled environments, only *Mammalia*-associated samples contained substantial phylogenetic novelty (Fig. 3a). Animal-associated samples were intermediate in terms of novelty, with a small number of substantial clades; human-associated samples contained almost no phylogenetic novelty of substantial abundance relative to other EMP biomes. The highest-ranked novel clade in *Mammalia*-associated samples was sister to the *Armatimonadetes* (previously OP10), a divergent lineage comprised of primarily environmental 16S rRNA gene sequences (Fig. 3c). Members of the *Armatimonadetes* are distributed across six groups, three of which consist entirely of environmental sequences (14). The three classes in this phylum are each monospecific and have diverse ecologies, including the rhizoplane of an aquatic plant (*Armatimonas rosea*) (15) and an isolate from a hydrothermal vent (*Chthonomonas calidirosea*) (16). The observed novel clade does not likely correspond to these habitats and is a considerably variable relative to these known reference sequences. This clade may represent a different related group of *Armatimonadetes* taxa associated with mammals, potentially expanding the number of sequence-based groups within the phylum. Notably, OP10 OTUs unclassified to family were observed in the rumen from a study of the gut microbiome of the North American moose (17). This is also consistent with the source of samples in the mammal-associated biome, a broad collection of fecal samples collected from mammals.

### Extreme ecosystems (cold/desert).

Cold environments (i.e., tundra, polar, and cold deserts and semideserts) contained substantial phylogenetic novelty. The largest divergent clade from cold environments was observed in the tundra ecosystem (Fig. 3d). This clade was sister to a large group of LTP reference sequences, mostly belonging to the *Chlorobiaceae* (green sulfur bacteria), *Cytophagaceae*, *Chitinophagaceae*, and *Flavobacteriaceae* (largest fraction). Due to substantial novelty and phylogenetic position as sister to the *Bacteroidetes*, this clade was further investigated as potentially uncharacterized organelles or chimerism. OTUs within this clade were largely classified as *Bacteria* only and had low identity to named representatives in BLASTn analysis. Fewer than 2% of sequences within the clade were putative chimeras as assessed against the RDP Gold library (18) using USEARCH/uchime (19). Furthermore, these sequences did not demonstrate significant BLASTn matches against *Rikenellaceae* or cyanobacterial sequences and therefore were unlikely to correspond to uncharacterized organelles.

Phylogenetic novelty was also broadly present in warm desert and semidesert samples, which included the most divergent clade in the analysis (Fig. 3a; supplemental material). Sequences from this clade had very low identity to GenBank sequences when excluding environmental and uncultured data (~82% identity to *Pelobacter propionicus* accession no. NR_074975). However, these sequences consistently matched environmental sequences from porous soils and soils from Madagascar (>95% identity). The largest novel clade (Fig. 3e) was also substantially divergent from reference sequences, and the closest LTP reference was *Acidothermus*, a monospecific genus that contains thermophilic, acidophilic, and cellulolytic species (20, 21).

### Terrestrial ecosystems.

Each of the various forested and grassland biomes contained a large number of novel clades (Fig. 3a). Forested ecosystems consistently contained the largest novel clades, with individual clades sometimes contributing 4 to 5% of all sequences in the biome (e.g., temperate broadleaf and tropical humid forests). Furthermore, deciduous forests (e.g., broadleaf and humid forests) tended to contain more uncharacterized novelty than the coniferous-dominated ecosystems (e.g., temperate needleleaf forests).

The temperate broadleaf biome contained the most abundant novel clade observed in any library, with ~5% of sequences. This clade was poorly ranked, despite its size, due to low relative phylogenetic novelty (mean phylogenetic distance of <0.2) and contained a small number of abundant OTUs that had *Burkholderia* spp. as the closest LTP references (supplemental data). Temperate broadleaf sites contained novel clades that were both abundant and divergent. For example, the highest-ranked clade (Fig. 3f) contained 1,403 OTUs sister to the *Bacteriovoraceae*, a family of predatory aerobic Gram-negative bacteria from a broad range of habitats (e.g., soil, marine, and freshwater). The finding of significant phylogenetic novelty in the temperate broadleaf biome (Fig. 3a and f) is consistent with the introduction of the family *Bacteriovoracaceae* (22). This family was further suggested to contain undefined species and genera due to the phylogenetic diversity of known taxa, which is consistent with the high number of unclassified OTUs observed in the highest-ranked clade recovered by SSUnique (Fig. 3f).

Tropical humid forests contained the largest number of abundant novel clades among the biomes studied, with five clades each contributing greater than ~2% total abundance, including multiple abundant clades with large mean phylogenetic distance. The highest-ranked clade was one of the most divergent clades identified in the study (Fig. 3g) and was also a substantial contributor to abundance (~2%). The closest LTP seed relative of this clade was *Streptomyces scabrisporus*, a soil isolate only “moderately related” to other *Streptomyces* species (23). This soil actinomycete produces the antibiotic hitachimycin (stubomycin), and the closest reference sequence was from a soil sample collected from a broadleaf forest at Ushiku-cho, Ibaragi Prefecture, Japan. These results reinforce that SSUnique-based analyses present a viable entry point into surveys of gaps in microbial taxonomy and studies of microbial dark matter (7). In addition to exploring uncharacterized phylogenetic novelty, SSUnique may be useful as a first step in taxonomy-directed bioprospecting surveys of habitats sampled in association with the EMP and similar survey efforts.

Grassland ecosystems (e.g., temperate or montane grasslands and terrestrial biomes) contained a large number of clades with substantial phylogenetic novelty, consistent with the known taxonomic richness of soil environments. However, these clades tended to be of low abundance (Fig. 3), in contrast with observations of SSUnique-processed data from forested sites.

### Human-associated ecosystems.

**FIG 4:**
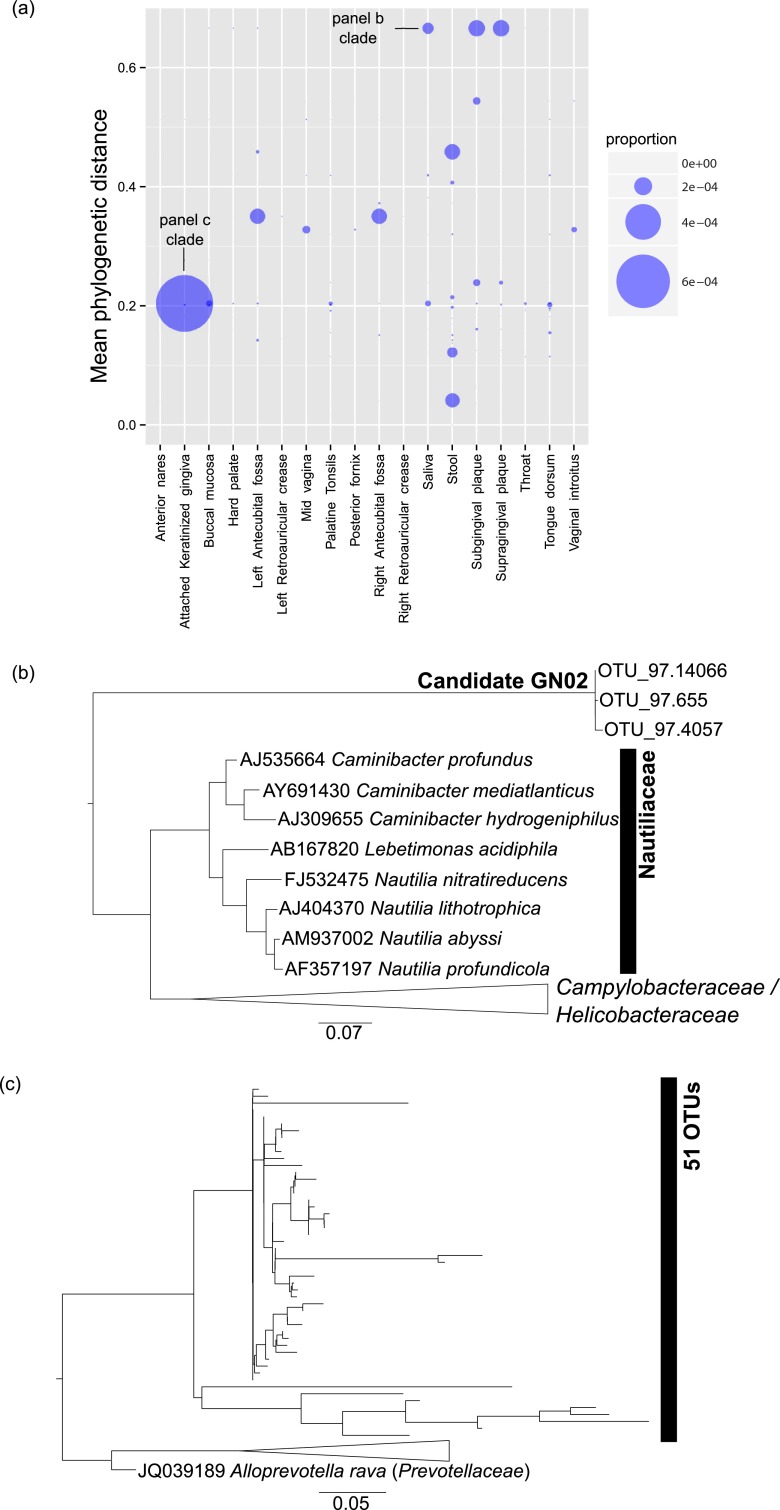
Phylogenetic novelty observed in the Human Microbiome Project. Novelty is partitioned across (a) body subsite, and representative novel clades were highlighted (with point size corresponding to the proportion of sequences in the library assigned to identified clade), including (b) the most divergent novel clade and (c) the most abundant novel clade.

Data from the HMP showed a substantial decrease in phylogenetic novelty, especially as a proportion of each library (Fig. 4). Generally, the highest phylogenetic novelty was observed in stool and oral sites. Despite the relatively low number of 151 novel groups identified by SSUnique, HMP data still contained contextually relevant novel clades. The relatively small number of novel OTUs in HMP data is likely a function of sequencing depth (i.e., fewer sequences per HMP sample) and careful data filtering (chimera detection, UCLUST [24], checks for contamination and mislabeling). Additionally, lower novelty in human-associated sites (EMP and HMP) relative to environmental samples and other animals likely reflects relatively low sample diversity and better existing characterization of the human microbiome. Contrasting human-associated novelty analysis in EMP and HMP data also demonstrates how the number of novel clades is influenced by the depth of sequencing and the sequence processing methods. Although this limits the ability to contrast counts and proportions across studies, it does not preclude using the method to highlight and identify phylogenetic novelty. The presence of sequences aligned with the candidate GN02 clade suggests substantial taxonomic blind spots even for relatively well-studied microbial communities, such as those associated with the human microbiome.

The most novel clades were observed in the stool, but the largest novel clades across all HMP samples were observed in oral sites, plaque, and saliva, and the most divergent novel clades were associated with attached gingiva (Fig. 4a). The most divergent clade contained roughly similar proportions of OTUs in the saliva, supragingival plaque, and subgingival plaque. This clade consisted of only three OTUs and was sister to a clade of thermophilic *Epsilonproteobacteria* (*Nautilaceae*) (Fig. 4b). Analysis of this clade with BLASTn identified sequences nearly identical to the candidate phylum GN02 (5), which is a group of bacteria identified through primer design against potential novelty in taxonomic reference data sets with the goal of supplementing human oral microbiome reference data (5). The OTUs aligned with this candidate phylum (Fig. 4b) suggest widespread occurrence of uncharacterized taxa in the oral microbiome and reinforce efforts to develop customized reference data sets.

The most abundant clade identified among HMP samples was observed in attached keratinized gingiva, which is the thick protective gum tissue surrounding the necks of teeth (Fig. 4c). Although this clade was not particularly novel, it was represented by >1,100 sequences (~0.06%) from this sampling site. This gingival sample was phylogenetically aligned with *Alloprevotella* sp. and *Prevotella* sp. from the feline (25) and canine (26) oral microbiome studies, respectively, both at ~94% identity (Fig. 4c). This clade was present but less abundant for other HMP mouth sites, variously rare in skin sites, and absent in stool and vaginal sites. This distribution suggests either a coevolved novel taxon associated with correlated human sites or microbial transfer between pets and cohabiting owners. The sequence identity patterns observed are intriguing, considering that human samples were equally divergent from feline and canine samples. A comparison of other mammalian oral microbiomes, both domesticated and wild, would provide a suitable test of these hypotheses.

Although SSUnique will unavoidably recover some sequencing and experimental artifacts as false-positive novel lineages if these artifacts are present in processed microbiome data (i.e., OTU tables), filtering for 16S rRNA secondary structure successfully removes many of these artifacts. Evidenced by more conservatively and consistently processed HMP data, in comparison to data processing by independent groups contributing to the EMP, the number of novel clades can be relatively small in some data sets. More stringent clustering and sequence processing approaches, such as UPARSE (27), result in far fewer novel clades relative to less stringent sequence processing. Regardless, even very conservatively processed microbiome data harbor substantial phylogenetic novelty (Fig. 4). Ranked novel clades and corresponding sequence alignments are available at https://github.com/neufeld/SSUnique/blob/master/supplemental.tar.gz.

### Conclusion.

The recovery of more phylogenetic novelty relative to reference data sets is perhaps unsurprising considering the magnitude of microbial dark matter (7) and the number of uncultivated microbial species captured in microbiome data. Despite a nontrivial amount of unclassified sequences corresponding to sequencing artifacts (28–32), it is clear that there exists untapped phylogenetic novelty in sequence repositories, and the automated approach shown here is an effective way to identify this novelty in microbiome data sets. Beyond observation and characterization of phylogenetic novelty as an end in itself, SSUnique provides an automated method for characterization of novel clades that can be used to screen for candidate ranks and, using extracted clade alignments, to improve curated reference data sets. Further genomic contexts can be explored by using novel clade profiles to search for corresponding contigs from metagenomic data. As sequence data continue to proliferate, tools such as SSUnique can be used to bridge microbiome and metagenomic studies of taxonomic blind spots and provide further context to environmental sequence data.

## MATERIALS AND METHODS

### Overview of SSUnique.

SSUnique is a pipeline implemented in the R programing language used for exploration of phylogenetic novelty in microbial ecology research. The goal of this pipeline is to identify monophyletic groups of unclassified operational taxonomic units (OTUs) in microbiome data, characterize observed phylogenetic novelty and genomic context, and provide data for downstream analyses. Broadly, the biom observation matrix (33) is filtered for OTUs that are unclassified at a specified rank (default, class). This subset is further filtered, removing potential sequence artifacts that do not conform to the 16S rRNA gene using ssu-align (11). Sequences for remaining OTUs are aligned using Infernal (34) and then merged with a reference alignment (e.g., Living Tree Project v.119 [13, 14]). The resulting alignment is used to construct a phylogeny for further analysis (default, FastTreeMP [35]). Tailored reference alignments can also be used, allowing for study-specific exploration (e.g., oral microbiome [36] or large subunit [LSU] rRNA). The resulting filtered OTUs are then grouped progressively from random terminal nodes into novel clades that are delineated by a clade containing a reference taxon (i.e., novel clades are defined as the largest monophyletic group sister to a clade containing a reference taxon). The resulting pool of novel clades is scored for novelty by averaging the ranked phylogenetic novelty (weighted mean branch length between novel terminal nodes and closest reference sequence) and ranked total abundance of OTUs within the clade. As a result, the highest-ranked clades will tend to be numerically abundant and contain the most phylogenetic novelty relative to reference sequences. Also included in the output are DNA profiles (37) constructed from the sequence alignment for each clade that can be used to identify metagenomic contigs corresponding to specified novel clades. This functionality provides downstream tools for targeted amplification, potential genomic context, and eventual markers for single-cell genomics. SSUnique also contains visualization tools for exploring phylogenetic novelty in microbiome data, especially useful for very large data sets. These include scaled bubble plots and density dot plots, partitioned by metadata that demonstrate novelty variation across categories.

### Availability.

SSUnique is implemented in R, requiring the ggplot2, BioStrings, reshape, and phyloseq (38) packages, and is available within AXIOME (39). Source code, install script, online wiki, and a sample workflow are available online at https://github.com/neufeld/SSUnique.

### Microbiome data.

To provide an initial benchmark of our automated analysis pipeline, a largely manual analysis of phylogenetic novelty within soils from Alert, NU (4), was reproduced using SSUnique. Subsequently, to maximize the observation of phylogenetic novelty and any underlying ecological patterns, we analyzed sequence and OTU data provided by the Earth Microbiome Project (EMP 10,000-snapshot OTU table [http://www.earthmicrobiome.org]) and the Human Microbiome Project (HMP; QIIME pipeline [http://www.hmpdacc.org/HMQCP/]), representing a broad distribution of habitats and environmental conditions. The EMP data consisted of 14,095 samples and 5,594,412 OTUs. The HMP data consisted of ~45,000 OTUs and >5,700 samples across 15 body sites (18 for female subjects). The EMP samples were separated by biome (ENV_BIOME metadata) to link phylogenetic novelty to sample metadata, separately analyzing major biome types, such as tundra, marine, and terrestrial. Human-associated data from both HMP and EMP were further separated by body site. To reduce spurious novel clades, very-low-abundance OTUs (<10 sequences) were removed independently from each analyzed subset. Each analysis, including parameters, data, and metadata scheme, is included within analysis scripts available with the source code.

### Analysis of phylogenetic novelty.

Each library was evaluated independently with initial filtering of OTUs unclassified to the rank of class. Using the SSUnique pipeline, sequence data corresponding to unclassified OTUs from each library were filtered for spurious sequences, retaining only those that conformed to a bacterial SSU model (ssu-align [11]). Generally, artifacts of OTU creation such as sequence chimerism and richness inflation should be encapsulated in OTU creation independent of the SSUnique pipeline. Advice for screening of such artifacts is included within the SSUnique documentation. Filtered, unclassified sequences were aligned to the SSU structural covariation model (cmalign/Infernal [12]) and merged with the LTP v.119 sequence data previously aligned by the same method (40, 41). An ML-approximate phylogeny was constructed using FastTreeMP (35) with the generalized time-reversible (GTR) model of nucleotide evolution. Novel clades demarcated by reference sequences were identified as outlined above and ranked for further characterization.

Phylogenetic novelty observed through the SSUnique pipeline may reflect data missing from the sequence reference backbone. To further establish potential novelty within the unclassified fraction (i.e., missing data in reference data), a 10% random subset for each EMP metadata category was used in a BLASTn analysis against the NCBI GenBank nonredundant (nr) nucleotide data set, excluding uncultured and environmental sequences. All ranked novel clades and corresponding sequence alignments are available in Table S1 and at https://github.com/neufeld/SSUnique/blob/master/supplemental.tar.gz.
