# Supplementary material for: SSUnique: Detecting Sequence Novelty in Microbiome Surveys
Source: mSystems. 2016 Dec 20;1(6):e00133-16. doi: 10.1128/mSystems.00133-16 (PMC5183599; doi:10.1128/mSystems.00133-16)
Supplement: Table S1 [file sys006162073st1.pdf]

**Suppl. Table 1.** Marker gene libraries and associated number of samples analyzed in this study.

| **Sequence set** | | **Description (metadata)** | **Number of Samples** | |
|---|---|---|---|---|
| Earth Microbiome Project (EMP 10,000) | | ENVO:animal-associated habitat | 1138 | |
| | | ENVO:aquatic biome | 936 | |
| | | ENVO:Cold-winter (continental) deserts and semideserts | 121 | |
| | | ENVO:freshwater biome | 426 | |
| | | ENVO:human-associated habitat | 2047 | |
| | | ENVO:mammalia-associated habitat | 1998 | |
| | | ENVO:marine biome | 938 | |
| | | ENVO:mediterranean forests, woodlands, and shrub biome | 1010 | |
| | | ENVO:mixed island systems | 127 | |
| | | ENVO:[M-m]ontane grasslands and shrubland biome | 240 | |
| | | ENVO:nest of bird | 627 | |
| | | ENVO:polar biome | 314 | |
| | | ENVO:[S-s]mall lake biome | 1990 | |
| | | ENVO:[T-t]emperate broadleaf and mixed forest biome | 174 | |
| | | ENVO:temperate coniferous forest biome | 19 | |
| | | ENVO:temperate grasslands<br>ENVO:[T-t]emperate grasslands, savannas, and shrubland biome | 839 | |
| | | ENVO:[T-t]emperate needle-leaf forests or woodlands | 33 | |
| | | ENVO:[T-t]errestrial Biome | 682 | |

| | | | | |
|---|---|---|---|---|
| | | ENVO:[T-t]ropical and subtropical moist broadleaf forest biome | 76 | |
| | | ENVO:tropical humid forests | 28 | |
| | | ENVO:tundra biome | 127 | |
| | | ENVO:warm deserts and semideserts | 97 | |
| Human Microbiome Project | | Human Microbiome Project (QIIME community profiling; http://www.hmpdacc.org/HMQCP/) | | The Human Microbiome Project Consortium, 2012 |
| Alert | | | 2 | Bartram et al. 2011, Lynch et al. 2012 |

* The EMP data set is particularly broad. Here we have used subsets of the data representing broad environmental classifications, excluding low-abundance or unclassified samples. ENVO:deserts and xeric shrubland biome (7), ENVO:large river biome (6), ENVO:mangrove biome (7), ENVO:marginal sea (8), ENVO:taiga (4), ENVO:tropical and subtropical coniferous forest biome (3), None (59). Low abundance OTUs (<10 sequences) were removed from analyses to minimize spurious novel clades.
